# Living with a rare disease: Why lived experience must shape research

**DOI:** 10.1371/journal.pmed.1005253

**Published:** 2026-09-18

**Authors:** Gemma Whyatt, Jodi Whitehouse

**Affiliations:** 1 Department of Public Health and Primary Care, University of Cambridge, Cambridge, United Kingdom; 2 Caring Matters Now, St Ives, United Kingdom

## Abstract

In this Perspective, Gemma Whyatt and Jodi Whitehouse—two women living with the rare condition Congenital Melanocytic Naevus—outline why lived experience must shape rare disease research from the outset, and discuss how genuine patient-researcher partnership transforms science, care and identity.

## Teaser

Rare diseases are often defined by numbers and clinical perspectives, a narrative that needs re-writing. Drawing on decades of patient advocacy, two women living with the rare condition Congenital Melanocytic Naevus argue that lived experience must shape research from the outset, and discuss how genuine patient-researcher partnership transforms science, care and identity.

Rare diseases are often defined by numbers. In Europe, a condition is considered rare when it affects fewer than or equal to 1 in 2,000 people [1]. Yet for the millions of individuals worldwide living with a rare disease, rarity is more than a statistic; it is a daily reality. It shapes childhood, healthcare experiences, identity, relationships, and hopes for the future.

**Fig 1:**
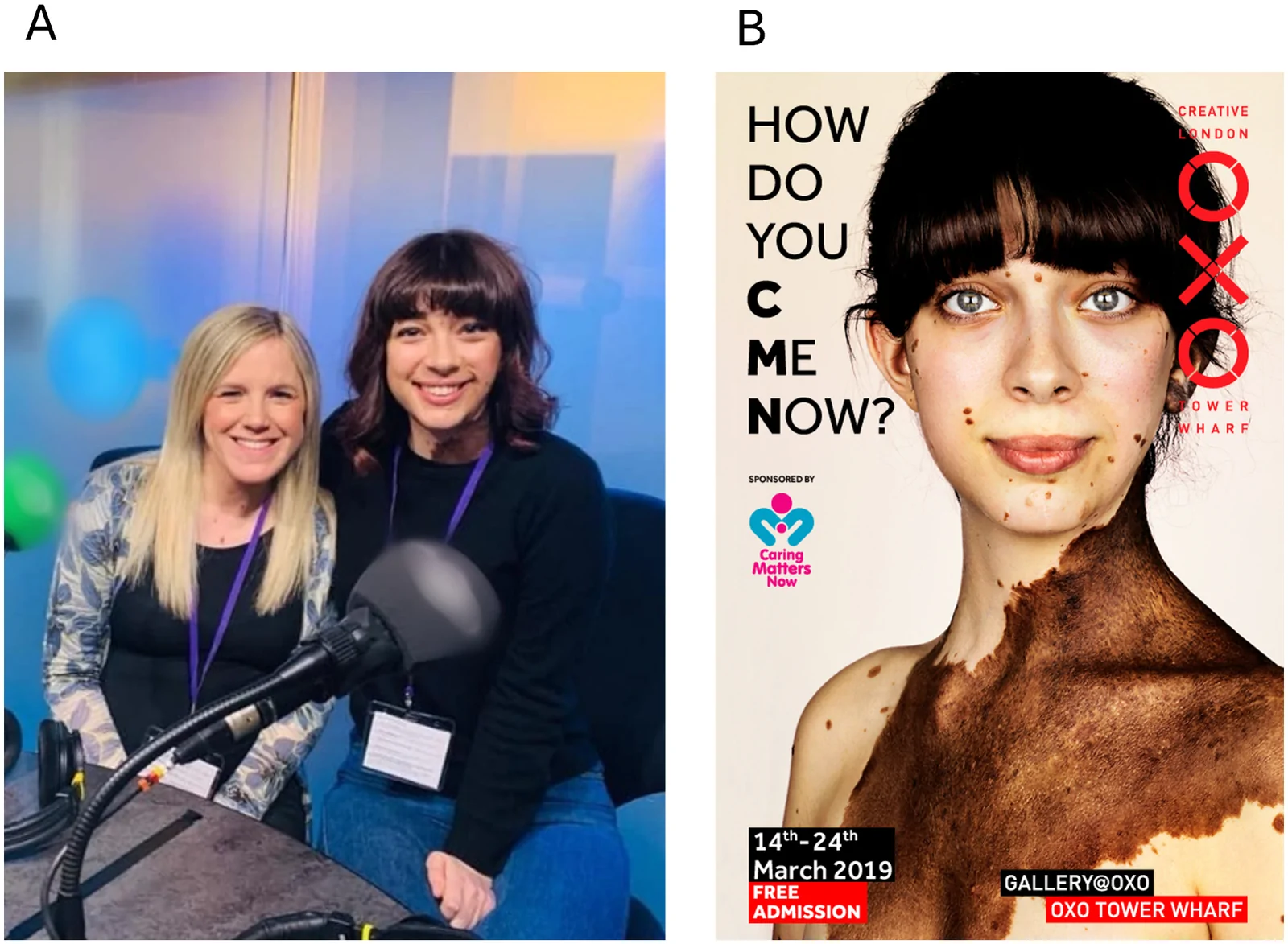
Raising awareness for rare diseases and patient advocacy. **(A)** Jodi (left) and Gemma (right) during a radio interview discussing the rare disease Congenital Melanocytic Naevus (CMN) in 2019 (Image used with permission from Gemma Whyatt). **(B)** Poster image for the How Do You C Me Now campaign for CMN, featuring Gemma’s portrait (Image used with permission from Jodi Whitehouse, Caring Matters Now, and photographer Brock Elbank).

We write this article from two different but interconnected perspectives: as patients living with Congenital Melanocytic Naevus (CMN), a rare genetic skin condition present at birth, and as advocates for patient-centred research and care. One of us (Jodi) founded a national patient organisation nearly three decades ago; the other (Gemma) became a clinician (Fig 1A). Together, we have witnessed how research can transform lives, but only when it is informed by the people it aims to serve.

## More than a diagnosis

CMN is a type of pigmented birthmark that is present at birth. These marks are often dark brown, hairy and raised. CMN can vary in size from small to very large, covering up to 80% of the body. For some individuals, CMN remains primarily a dermatological condition. For others, it can be associated with neurological complications and an increased risk of melanoma. The severity, visibility and impact vary considerably [2].

For many families, however, the first experience is remarkably similar. A baby is born and healthcare professionals gather around. Parents quickly realise that something is different. Questions are asked that no one can answer. Frequently, they hear words that no parent wants to hear: *“I’ve never seen this before”.*

One of us, Jodi, was born with CMN covering approximately 80% of her body. During childhood, she underwent more than 30 surgical procedures and missed several years of schooling. Doctors had little understanding of the condition at the time. The dominant clinical response was intervention, often driven by uncertainty rather than evidence [3]. It was not until age 16, after meeting Dr David Atherton at Great Ormond Street Hospital, that she was given a name for the condition she had lived with her entire life.

The significance of a diagnosis should never be underestimated. A diagnosis does not simply categorise a condition. It provides language, understanding and a starting point for connection. For rare disease patients, being able to name a condition is often the first step away from isolation.

## The burden of visibility and invisibility

Rare diseases are often discussed from a clinical perspective, yet many of their greatest impacts are psychosocial [4]. For those living with visible conditions, difference is frequently noticed before identity is recognised. Strangers stare. Questions are asked in public. Assumptions are made. Children quickly learn that they look different from their peers.

Gemma describes her childhood as feeling *“medicalised before it was mine”.* Born with extensive CMN, she also underwent multiple surgical procedures from an early age. As she grew older, she became more aware of her differences and the psychosocial impact this had on her. Yet the visibility of rare diseases is not unique in creating these challenges. Whether conditions are visible or invisible, affected individuals often describe similar experiences: misunderstanding, social isolation, uncertainty and a lack of recognition [5]. The rarity itself can be as burdensome as the disease. Patients frequently become experts in their own condition because few others around them understand it.

The cumulative effect of these experiences can shape confidence, self-image, educational opportunities, employment and mental wellbeing far into adulthood. These outcomes are rarely captured fully in clinical datasets, but they are central to the lived experience of rare disease.

One of the most transformative moments for many rare disease patients is meeting someone else who shares their condition. When Jodi was 16, Dr Atherton asked if he could share her family’s home telephone number with another family affected by CMN. That single conversation led to a gathering of eight families in a church hall in Liverpool. Jodi’s grandparents served tea and homemade cake while families shared stories, fears and experiences. What began as a small support meeting eventually became Caring Matters Now, which now supports more than 900 families across the United Kingdom and internationally.

For Gemma, meeting others with CMN was life-changing. *“For the first time”,* she recalls, *“I wasn’t a medical anomaly. I was part of a community”.*

Community does more than provide emotional support. It creates belonging. It provides context for experiences that may otherwise feel overwhelming. It allows children to see adults thriving with the condition they themselves are growing up with. It transforms isolation into possibility.

## Changing the narrative

In 2019, Caring Matters Now launched “*HOW DO YOU C ME NOW?”*, a global photographic exhibition featuring 30 children and adults living with CMN from 13 countries across five continents (Fig 1B). The exhibition attracted more than 8,000 visitors in a single week and generated national and international media attention. Its purpose was simple: to challenge perceptions.

The results were striking. Individuals photographed reported feeling empowered by publicly sharing their stories, expressing increased confidence and acceptance of their appearance. Visitors reported more positive attitudes toward visible differences after viewing the exhibition [6]. For many participants, the project represented a shift from being defined by their condition to owning their narrative. The exhibition demonstrated something healthcare systems sometimes forget: when people see difference through a human lens rather than a medical one, understanding grows.

## When patients and researchers become partners

One of the most important lessons we have learned is that patients should not merely participate in research; they should help shape it.

Nearly three decades ago, Caring Matters Now began working closely with Professor Veronica Kinsler and colleagues at Great Ormond Street Hospital and the Francis Crick Institute to better understand CMN. What started as conversations with families evolved into one of the most significant patient-research partnerships in rare dermatology, which helped identify the genetic causes of CMN, establish evidence-based care pathways, improve patient information and advance therapeutic research [7].

In our experience, partnerships like this take several concrete forms. Patients and families help set research priorities, pushing questions beyond narrow clinical risk (in CMN’s case, melanoma) toward outcomes that shape everyday life, such as psychosocial impact, scarring and time lost from school. They help choose which outcomes are measured in the first place; initiatives such as the OCOMEN project, which brought patients, parents and clinicians together to define a core outcome set for CMN, show how patient and parent input can determine what counts as a meaningful outcome in clinical care and research, not clinicians alone [8]. These partnerships also enable recruitment and sustained engagement: the trust that patient organisations such as Caring Matters Now build over decades is often what allows families to contribute biological samples, commit to long-term follow-up, and take part in initiatives like this at all. None of these are incidental contributions. Setting priorities, choosing outcomes and building the trust that enables recruitment are forms of scientific labour in their own right.

Successes of such collaborations are evident across rare disease research. Patient and public involvement has, for example, been formalised in the James Lind Alliance Priority Setting Partnership for epidermolysis bullosa, where patients, carers and clinicians jointly ranked the research questions that mattered most to those living with the condition [9]. In Duchenne muscular dystrophy, sustained parent-led advocacy has shaped regulatory guidance on patient-focused drug development, ensuring that outcomes meaningful to families, not only biomarkers, inform how new treatments are evaluated [10]. Patient registries co-designed with those affected, such as the UK Cystic Fibrosis Registry and Enroll-HD in Huntington’s disease, show how the same kind of sustained partnership can accelerate research and shape access to care in very different conditions [11,12]. The clinical specifics vary; the underlying practice does not.

## Looking forward

The landscape for CMN has changed dramatically over the past 30 years. Today there is greater clinical understanding, established specialist services, international patient networks and promising scientific developments that were unimaginable when Caring Matters Now was founded. Yet, the lessons extend beyond CMN.

Our message to clinicians, researchers, policymakers and healthcare leaders is simple: Behind every sample is a person, behind every dataset is a family, and behind every research question is someone waiting for clarity about their future.

Scientific discovery is powerful, but it becomes extraordinary when guided by lived experience. If we want research to achieve its greatest impact, patients must not sit at the edge of the process. While researchers study the gene, patients live the phenotype. Both perspectives are essential, and only when patients are involved can we ensure that advances in science improve not only health outcomes, but also confidence, identity and quality of life for those living with rare diseases every day.

## Abbreviation:

CMN: Congenital Melanocytic Naevus

## References

[1] Wang CM, Whiting AH, Rath A, Anido R, Ardigò D, Baynam G, et al. Operational description of rare diseases: a reference to improve the recognition and visibility of rare diseases. Orphanet J Rare Dis. 2024;19(1):334. doi: 10.1186/s13023-024-03322-7 39261914 PMC11389069

[2] Kinsler VA, O’Hare P, Bulstrode N, Calonje JE, Chong WK, Hargrave D, et al. Melanoma in congenital melanocytic naevi. Br J Dermatol. 2017;176(5):1131–43. doi: 10.1111/bjd.15301 28078671 PMC5484991

[3] Kinsler VA, Bulstrode N. The role of surgery in the management of congenital melanocytic naevi in children: a perspective from Great Ormond Street Hospital. J Plast Reconstr Aesthet Surg. 2009;62(5):595–601. doi: 10.1016/j.bjps.2008.12.016 19244003

[4] Fenech G, Fondacaro DV, New KJ. Mental health challenges and psychosocial impact of rare diseases in children and adolescents -a systematic review. J Rare Dis. 2026;5(1):67. doi: 10.1007/s44162-026-00207-0

[5] Bolz-Johnson M, Johnson K, Rietman A, Baynam G, Kwast Hoekstra D, Grano C, et al. The psychological needs of people living with a rare condition [Internet]. EURORDIS-Rare Diseases Europe; 2026 Apr [cited 2026 Aug 7]. Available from: https://www.eurordis.org/publications/the-psychological-needs-of-people-living-with-a-rare-condition-results-of-a-literature-review/.70790/AXUH4676

[6] Zolkwer MB, Whitehouse J, Sanderson SC, Kinsler VA. Impact of public exhibition on the perception of birthmarks. Pediatr Dermatol. 2023;40(4):615–20. doi: 10.1111/pde.15315 37212633

[7] Kinsler VA, O’Hare P, Jacques T, Hargrave D, Slater O. MEK inhibition appears to improve symptom control in primary NRAS-driven CNS melanoma in children. Br J Cancer. 2017;116(8):990–3. doi: 10.1038/bjc.2017.49 28253523 PMC5396107

[8] Fledderus AC, Pasmans SGMA, Wolkerstorfer A, Oei W, Etchevers HC, van Kessel MS, et al. Domains and outcomes of the core outcome set of congenital melanocytic naevi for clinical practice and research (the OCOMEN project): part 2. Br J Dermatol. 2021;185(5):970–7. doi: 10.1111/bjd.20437 33959942 PMC9290785

[9] UK DEBRA. Global EB priority setting partnership: identifies research priorities for the four main types of epidermolysis bullosa. Bracknell, UK: DEBRA; 2025. Available from: https://www.debra.org.uk/wp-content/uploads/2025/06/DEBRA-Priority-setting-partnership-JLA-results-booklet-digital.pdf

[10] Furlong P, Bridges JFP, Charnas L, Fallon JR, Fischer R, Flanigan KM. How a patient advocacy group developed the first proposed draft guidance document for industry for submission to the U.S. Food and Drug Administration. Orphanet J Rare Dis. 2015;10:82. doi: 10.1186/s13023-015-0281-2 PMC4486430 26104810

[11] Taylor-Robinson D, Archangelidi O, Carr SB, Cosgriff R, Gunn E, Keogh RH, et al. Data resource profile: the UK Cystic Fibrosis Registry. Int J Epidemiol. 2018;47(1):9–10e. doi: 10.1093/ije/dyx196 29040601 PMC5837577

[12] Landwehrmeyer GB, Fitzer-Attas CJ, Giuliano JD, Gonçalves N, Anderson KE, Cardoso F, et al. Data analytics from Enroll-HD, a global clinical research platform for Huntington’s disease. Mov Disord Clin Pract. 2016;4(2):212–24. doi: 10.1002/mdc3.12388 30363395 PMC6174428

